# Albumin excretion rate among patients with diabetic retinopathy

**DOI:** 10.22088/cjim.11.2.177

**Published:** 2020

**Authors:** Makbul Aman, Haerani Rasyid, Suriana Dwi Sartika, Himawan Sanusi, Hasyim Kasim, Syakib Bakri, Muhammad Ichsan, Arifin Seweng

**Affiliations:** 1 Division of Endocrine Metabolic and Diabetes, Department of Internal Medicine, Faculty of Medicine, University of Hasanuddin, Makassar, Indonesia; 2 Division of Nephrology, Department of Internal Medicine, Faculty of Medicine, University of Hasanuddin, Makassar, Indonesia; 3 Department of Internal Medicine, Faculty of Medicine, University of Hasanuddin, Makassar, Indonesia; 4 Department of Ophtalmology, Faculty of Medicine, University of Hasanuddin, Makassar, Indonesia; 5 Department of Biostatistics, Faculty of Public Health, University of Hasanuddin, Makassar, Indonesia

**Keywords:** Diabetes mellitus type 2, Albumin excretion rate, Diabetic retinopathy

## Abstract

**Background:**

Chronic microvascular complications consist of diabetic nephropathy (DN), diabetic retinopathy (DR), and diabetic neuropathy. Diabetic nephropathy is assessed through albuminuria, and diabetic retinopathy is assessed through fundoscopy. Several studies have assessed the albuminuria in diabetic retinopathy but have found inconclusive results. This study aims to investigate the albumin excretion rate in patients with diabetic retinopathy.

**Methods:**

A cross sectional design was applied in this study. The diagnosis of type 2 diabetes mellitus was determined based on the anamnesis and laboratory examinations. The study was conducted at Dr. Wahidin Sudirohusodo Hospital and Hasanuddin University Hospital in Makassar during November 2018 until April 2019. The stages of diabetic retinopathy were based on funduscopic examinations. In addition, the blood pressure, BMI, albumin excretion rate, lipid profile, and HbA1C were also examined. *Chi Square* and *Kappa* tests were performed in the statistical analysis.

**Results:**

120 subjects with type 2 diabetes mellitus were observed. Of the total subjects, the number of females within the age of 36-79 years made up the biggest fraction. There was a significant relation between hypertension comorbidity with the albumin excretion rate and grading diabetic retinopathy where the A3 and proliferative diabetic retinopathy (PDR) percentages were higher in the hypertension group at 68.8% and 54.5%. There was also a significant correlation between incidence of albuminuria with diabetic retinopathy. Particularly, proliferative diabetic retinopathy (PDR) remained associated with albuminuria, while non-proliferative diabetic retinopathy (NPDR) was related to non-albuminuria.

**Conclusion:**

Albuminuria incidence confirms association with diabetic retinopathy grading.

The incidence and prevalence of diabetes mellitus (DM) have increased globally, especially in the big cities, including in Indonesia (1, 2). The growth of the incidence of diabetes mellitus is certainly followed by the acceleration of chronic complications of DM. Numerous prospective studies clearly indicate that occlusion of blood vessels in both microvascular and macrovascular aggravates any disease (2). Chronic microvascular complications of diabetes mellitus are diabetic nephropathy (DN), diabetic retinopathy (DR), and diabetic neuropathy. Diabetic nephropathy is the leading cause of renal failure which requires renal replacement therapy, while diabetic retinopathy is the leading cause of blindness in patients with diabetes (3, 4). Several studies have assessed the albumin excretion rate in diabetic retinopathy. Studies by Pavel showed that the rate of renal damage had a proportional association with the level of eye damage in patients with type 2 diabetes mellitus (T2DM). 

In this study, the grade of renal impairment was assessed by urinary albumin excretion and the rate of eye damage assessed from proliferative or non-proliferative forms. Another study by JDCS (Japan Diabetes Complications Study) during 8 years of follow-up showed the severity of retinopathy correlated to the decrease in glomerular filtration rate and progression microalbuminuria to macroalbuminuria. However, two of these studies are not in line with the research by June Won Lee et al, which found that proliferative diabetic retinopathy was correlated only with microalbuminuria and not with macroalbuminuria (5–7). From the aforementioned differences, this research aims to assess the albuminuria among patients with diabetic retinopathy.

## Methods

The research was conducted during November 2018 - April 2019 at two hospitals: Dr Wahidin Sudirohusodo Hospital and Hasanuddin University Hospital. A cross-sectional study was performed on the samples of T2DM patients who met the inclusion criteria. Those with the following criteria were included: (1) >18 years old (2) >5 years with DM (3) willing to participate in the research. On the other hand, the exclusion criteria included (1) severe infection (2) pregnancy (3) malignancy (4) heart failure (5) hematuria (6) using an angiotensin converting enzyme (ACE) inhibitor or angiotensin receptor blocker (ARB). The diagnosis of T2DM was based on the history and physical examination. 

The form of consent was given to each patient who participated as a research sample. Subsequently, the administrative agreement was issued by the ethical requirements of the Commission of Biomedical Research on Humans, Faculty of Medicine, Universitas Hasanuddin.

Diabetic retinopathy assessment was divided into non-proliferative and proliferative. Albuminuria is divided into A1 (<30 µg/mg), A2 (30-300 µg/mg), and A3 (> 300 µg/mg). HbA1C were divided into controlled (HbA1c <7) and uncontrolled (HbA1C≥ 7). Assessment of blood pressure was classified into hypertensive and non-hypertensive. Levels of lipid profile were reported in the form of quantitative (mg/dl). Body mass index (BMI) was categorized into underweight, normal, overweight, obese I, and obese II. Statistical analysis applied in this research was the distribution of frequencies, *Chi Square* and *Kappa* tests. The approximate samples were calculated by the formula as follows:

 n = NZ^2^P(1-P)                 d^2^(N-1) + Z^2^ P (1-P)

N = Estimated study population of 200 

Z = standard deviation of normal value (1.96)

P = Estimated proportion of surveyed attribute 0.500

d = degree of accuracy to be assessed =0.07

The application of the formula resulted in a minimum of 100 samples. The possibility of dropped-out subjects was measured through the calculation as follows:

 100+ (100x10%)=110. 

As a result, a minimum of 110 subjects were eligibly selected. 

## Results

Data analysis was conducted on 120 subjects with T2DM within the age of 36-79 years, with a mean of 55 ± 9 years. Table 1 describes the subject characteristics of this study, consisting of gender, BMI, blood pressure, HbA1C, lipid profile, albuminuria, and funduscopy. Subjects consisted of men (36.7%) and women (63.3%). 

**Table 1:** Characteristics of Subjects

**Variables**		**n**	**%**
Gender	Male	44	36.7
	Female	76	63.3
BMI	Normal	35	29.2
	overweight	34	28.3
	obese 1	41	34.2
	obese 2	10	8.3
Blood pressure	Hypertension	80	66.7
	Non hypertension	40	33.3
HbA1c	controlled	8	7.4
	Not controlled	100	92.6
Cholesterol	Normal	19	15.8
	High	101	84.2
LDL	Normal	16	13.3
	High	104	86.7
HDL	Low	31	25.8
	Normal	89	74.2
TG	Normal	47	39.1
	High	73	60.9
Albuminuria	A1	22	18.3
	A2	29	24.2
	A3	69	57.5
Funduscopic	Normal	6	5.0
	NPDR	55	45.8
	PDR	59	49.2

LDL= low density lipoprotein; HDL= high density lipoprotein TG= triglyceride; BMI= body mass index; NPDR= non proliferative diabetic retinopathy; PDR= proliferative diabetic retinopathy

According to BMI, 70.8% of the subjects were identified as overweight or obese. Most of the research subjects had hypertension and uncontrolled HbA1C levels with 66.7% and 92.6% respectively. Based on lipid profile, the majority of the research subjects were identified as high cholesterol (84.2%), normal HDL level (89%), high TG level (60.9%), and high LDL level (86.7%). On the other hand, the prevalence of albuminuria was 18.3% for A1, 24.2% for A2, and 57.5% for A3. Based on the grade of retinopathy, the prevalence constituted 5% normal, 45.2% of NPDR, and 49.2% of PDR. There was no significant difference in the grade of albuminuria among gender, age, BMI, HbA1c, total cholesterol, triglyceride levels, HDL, and LDL (p>0.05). Table 2 describes a significant correlation between the hypertension comorbidity with albuminuria, in which A3 percentage is higher in the hypertension group (68.8%) compared with non-hypertension (35.0%). Table 3 shows no significant relationship between the severity of diabetic retinopathy and the characteristics on gender, age, BMI and HbA1c, total cholesterol, and triglyceride levels (p>0.05).

Furthermore, a significant association was found between blood pressure and a grading of diabetic retinopathy (p<0.05) in which the percentage of PDR was found significantly higher in hypertension group compared with the non-hypertension with 57.5% compared and 32.5% respectively (p<0.05). 

Based on the level of LDL, the percentage of PDR was found significantly higher in the high LDL (p>0.05). Based on the levels of HDL, the percentage of PDR was identified significantly higher in HDL lower than in the normal, ie 71.0% compared to 41.6% (p<0.01). On the metabolic characteristic blood pressure has more significant relation to albuminuria rate (P=0.002) compared with diabetic retinopathy (p=0.034). 

**Table 2:** Correlations of gender, age, and metabolic factors with albuminuria

		Albuminuria, N (%)			p
		A1	A2	A3	
**Gender**	Man	4 (9.1)	14 (31.8)	26 (59.1)	0.084
	Woman	18 (23.7)	15 (19.7)	43 (56.6)	
**Age**	<65 years	19 (18.6)	24 (23.5)	59 (57.8)	0.923
	≥ 65 years	3 (16.7)	5 (27.8)	10 (55,6)	
**BMI**	Normal	6 (17.1)	9 (25.7)	20 (57.1)	0.640
	overweight	4 (11.8)	10 (29.4)	20 (58.8)	
	obese 1	11(26.8)	7 (17.1)	23 (56.1)	
	obese 2	1 (10.0)	3 (30.0)	6 (60.0)	
**Blood pressure**	Non Hypertension	12 (30.0)	14 (35.0)	14 (35.0)	0.002
	Hypertension	10 (12.5	15 (18.8)	55 (68.8)	
**HbA1C**	controlled	1 (12.5)	0 (0.0)	7 (87.5)	0.102
	Not controlled	21 (21)	29 (29)	50 (50)	
**Total** **Cholesterol**	Normal	4 (21.1)	5 (26.3)	10 (52.6)	0.928
	High	19 (18.8)	24 (23.8)	58 (57.4)	
**LDL**	Normal	2 (12.5)	5 (31.3)	9 (56.3)	0.694
	High	20 (19.2)	24 (23.1)	60 (57.7)	
**Triglycerides**	Normal	11 (23.9)	10 (21.7)	25 (54.3)	0.364
	High	11 (14.9)	19 (25.7)	44 (59.5)	
**HDL**	Low	4 (12.9)	7 (22.6)	20 (64.5)	0.585
	Normal	18 (20.2)	22 (24.7)	49 (55.1)	

BMI = body mass index; LDL = low density lipoprotein; HDL = high density lipoprotein,

**Table 3:** Correlations of gender, age, and metabolic factors with diabetic retinopathy

		**Diabetic retinopathy, N (%)**			**p**
		Normal	NPDR	PDR	
**Gender**	Man	1 (2.3)	19 (43.2)	24 (54.5)	0.461
	woman	5 (6.6)	36 (47.4)	35 (46.1)	
**Age**	<65 years	4 (3.9)	44 (43.1)	54 (52.9)	0.099
	≥ 65 years	2 (11.1)	11 (61.1)	5 (27.8)	
**BMI**	Normal	0 (0.0)	17 (48.6)	18 (51.4)	0.258
	overweight	1 (2.9)	12 (35.3)	21 (61.8)	
	obese 1	4 (9.8)	21 (51.2)	16 (39.0)	
	obese 2	1 (10.0)	5 (50.0)	4 (40.0)	
**Blood pressure**	Non-hypertension	3 (7.5)	24 (60.0)	13 (32.5)	0.034
	Hypertension	3 (3.8)	31 (38.8)	46 (57.5)	
**HbA1C**	controlled	0 (0.0)	4 (50.0)	4 (50.0)	0.770
	Not controlled	6 (6.0)	49 (49.0)	45 (45.0)	
**Total** **Cholesterol**	Normal	2 (10.5)	6 (31.6)	11 (57.9)	0.255
	High	4 (4.0)	49 (48.5)	48 (47.5)	
**LDL**	Normal	3 (18.8)	9 (56.3)	4 (25.0)	0.008
	High	3 (2.9)	46 (44.2)	55 (52.9)	
**Triglycerides**	Normal	4 (8.5)	17 (36.2)	25 (54.3)	0.155
	High	2 (2.7)	38 (51.4)	34 (45.9)	
**HDL**	Low	0 (0.0)	9 (29.0)	22 (71.0)	0.013
	Normal	6 (6.7)	46 (51.7)	37 (41.6)	

BMI = body mass index; LDL = low density lipoprotein ; HDL = high density lipoprotein, NPDR= non proliferative diabetic retinopathy; PDR= proliferative diabetic retinopathy

Table 4 shows that there was a significant association between albuminuria and diabetic retinopathy (p<0.05), in which PDR remains associated with albuminuria and NPDR is related to non-albuminuria.

Table 5 indicates that there is no significant association between albuminuria and the grading of diabetic retinopathy (p>0.05). However, the tendency of PDR is found the highest on the A3 (59.4%) and the lowest in A1 (22,7%), while the highest percentage of NPDR is on A1 (72.7%), and the lowest is on A3 (36.2%). 

**Table 4:** Correlations of genesis albuminuria with diabetic retinopathy grading

Albuminuria	diabetic retinopathy		p
	NPDR N(%)	PDR N(%)	
**Non-albuminuria**	16(76.2)	5(23.8)	0.005
**Albuminuria**	39(41.9)	54(58.1)	

NPDR= non proliferative diabetic retinopathy; PDR = proliferative diabetic retinopathy

**Table 5:** Correlations between the grading of albuminuria and diabetic retinopathy

Albuminuria	Diabetic retinopathy, N (%)			p
	Normal	NPDR	PDR	
A1	1 (4.5)	16 (72.7)	5 (22.7)	0.102
A2	2 (6.9)	14 (48.3)	13 (44.8)	
A3	3 (4.3)	25 (36.2)	41 (59.4)	

NPDR: non proliferative diabetic retinopathy; PDR: proliferative diabetic retinopathy

## Discussion

This study reported that grading of albuminuria had no remarkable correlation with severity of diabetic retinopathy (p>0.05). However, this study reported that there was a significant association between the incidence of albuminuria and diabetic retinopathy (p<0.05), where PDR remained associated with albuminuria (A2 or A3), and NPDR indicated a significant correlation with non-albuminuria. The insignificant correlation can be resulted from other metabolic factors that affect the rate of retinopathy instead of the albuminuria. In addition, the concept of non-proteinuria diabetic renal disease has been discussed in the last several decades in which the decline in glomerular filtration rate can occur without albuminuria.

 About one-third to one-half of patients with T2DM with reduced glomerular filtration rate did not have proteinuria, in which the level of renal damage in diabetic nephropathy was not eligible to be evaluated by the albuminuria (7). This study in line with the study by Boelter et al. that PDR had a significant relationship to microalbuminuria (8). Another study by Manaviat et al. showed that microalbuminuria had a correlation to the PDR and could be used as a marker of the risk of PDR (9). 

Diabetic retinopathy and nephropathy showed an association with the progression of endothelial dysfunction with parallel relation (10). When the advanced stages of retinopathy occurred, there was an alteration on glomerular histology and increased excretion of protein (11). Activation of metabolic factors (polyol pathway, increased AGE, protein kinase C (PKC) formation, oxidative stress, and inflammatory mechanisms play a vital role in the progression of diabetic retinopathy and nephropathy. 

Hyperglycemia causes endothelial damage, thickening of the basement membrane, platelet aggregation, retinal barrier breakdown, and the adhesion of leukocytes to the retinal capillaries. The presence of chronic hypoxia in diabetes induces several factors of angiogenic of growth factor that produces neovascularization in the retina and is called proliferative diabetic retinopathy. In diabetic nephropathy, hyperglycemia also induces the expression of growth factors and cytokines cause imbalance of glomerular cell and resulting in ultimately thickened tubular and glomerular basement membrane, mesangial matrix accumulation, and albuminuria (12–14). 

 In this research, the presence of comorbid hypertension was correlated with the albuminuria and grading of diabetic retinopathy. This is in line with the study by Ahmed et al. showing that the presence of comorbid hypertension had an association with the incidence of diabetic retinopathy (15). Blood pressure control is required to inhibit the progression of retinopathy and diabetic nephropathy. UK Prospective Diabetes Study (UKPDS) trial showed that blood pressure control could manage the progression of retinopathy by 47%. A research by Dhafer et al. indicated that poor control of blood pressure was associated with high levels of albuminuria in diabetes mellitus (16, 17). Increased blood pressure has an important role in the pathogenesis of diabetic retinopathy and nephropathy. It causes oxidative stress and inflammation, and has effect on blood flow, causing endothelial damage and contributing to the progression of diabetes complications (18). 

The mechanism of hypertension affecting the progression of diabetic retinopathy is a disturbed hemodynamic mechanism (autoregulation and hyperperfusion). Hypertension cause disturbed hemodynamic mechanism (autoregulation and hyperperfusion) and leads upregulation of VEGF expression in the endothelial cells of the retina (18). Hypertension is a major risk factor in the development and progression of diabetic nephropathy. It causes an increased intraglomerular pressure. Dilatation of the afferent glomerular accommodates the transmission of systemic blood pressure into glomerular. On the other hand, hyperglycemia causes disruption of the glomerular microcirculation autoregulation thereby worsening the effect of blood pressure in the glomerulus. Furthermore, hypertension induces stretching intraglomerular mesangial cells, resulting in excessive production of extracellular matrix and cytokines that aggravate diabetic nephropathy (19, 20).

In conclusion, blood pressure in albuminuria was significantly associated with excretion rate (p=0.002) rather than diabetic retinopathy (p=0.034). This is because hypertension without diabetes mellitus can cause an increase in albuminuria. Consequently, comorbid hypertension in diabetes mellitus increase the value of albuminuria. However, in diabetic retinopathy, hypertension causes hypertensive retinopathy in which funduscopic description is different from diabetic retinopathy.

This study confirms that there is a significant correlation between albuminuria incidence and grading of diabetic retinopathy. Albuminuria can be used to predict proliferative diabetic retinopathy and the progression of diabetic retinopathy. 

Therefore, it is suggested that follow-ups on the levels of albuminuria among patients with T2DM are necessary. This study cannot be used to determine the effect of lipid profile levels and HbA1C values on albuminuri and the severity of diabetic retinopathy. We did not include the medication such as medication for diabetes, lipid-lowering, and triglyceride-lowering medicines. 
